# The TP73 Gene Polymorphism (rs4648551, A>G) Is Associated with Diminished Ovarian Reserve

**DOI:** 10.1371/journal.pone.0120048

**Published:** 2015-03-20

**Authors:** Laura Diniz Vagnini, Adriana Renzi, Gabriela Ravanelli Oliveira-Pelegrin, Maria do Carmo Tomitão Canas, Claudia Guilhermino Petersen, Ana Lucia Mauri, João Batista Alcantara Oliveira, Ricardo Luiz Razera Baruffi, Mario Cavagna, José Gonçalves Franco Junior

**Affiliations:** 1 Paulista Center for Diagnosis, Research and Training, Ribeirão Preto, São Paulo, Brazil; 2 Center for Human Reproduction Prof. Franco Jr, Ribeirão Preto, São Paulo, Brazil; Zhejiang University, CHINA

## Abstract

It’s known that the members of the TP53 family are involved in the regulation of female reproduction. Studies in mice showed that the TP73 gene (member of this family) plays a role in the size of follicular pool, ovulation rate and maintenance of genomic stability. In the present study we analyzed data from 605 patients with ≤ 37 years attending their first intracytoplasmic sperm injection (ICSI). The association between the TP73 polymorphism (rs4648551, A>G) and the following parameters related to ovarian reserve, like age, antral follicular count (AFC), anti-Mullerian hormone levels (AMH) and ovarian response prediction index (ORPI) was evaluated. Our results showed an association of the AA genotype with diminished ovarian reserve (AMH <1, AFC ≤9). Women presenting the AA genotype had a 2.0-fold increased risk for having AMH <1 and AFC ≤9 (OR 2.0, 95% CI 1.23-3.31, P = 0.005). Patients presenting AA genotype had the lowest levels of AMH (P = 0.02), the lowest number of antral follicles (P = 0.01) and the lowest ORPI (P = 0.007). Analyzing the alleles, we can see an enrichment of the A allele in the group of diminished ovarian reserve (OR 1.4, 95%CI 1.02-1.83, P = 0.04). To the best of our knowledge, the present study is the first to analyze this polymorphism in humans for assessing the numbers of ovarian follicles and AMH levels and, therefore, the ovarian reserve. Our findings can contribute to the use of this polymorphism as a potential marker of diminished ovarian reserve.

## Introduction

Current studies show that about 17% of couples are unable to conceive and their infertility could stem from different causes, genetic factors representing a significant component [1–3]. The members of the p53 family (p63, p73 and p53), besides their roles in cell cycle regulation, transactivation and apoptosis, are involved in female reproduction and blastocyst implantation [4]. The p53, p63 and p73 are powerful transcription factors, and small variations in their activation status can cause modifications on the expression pattern of genes that they transcribe, modifying the fate of the cell [5].

Studies in mice have showed that the p73 gene plays a role in female infertility. The p73 gene contains two promoters that drive the expression of two major groups of p73 isoforms with opposing cellular actions: pro-apoptotic and anti-apoptotic [6, 7] and both are required for normal embryogenesis [8]. Mice lacking either one of the groups of isoforms are infertile, but the root of the infertility differ [9]. Mice that are deficient for both p73 groups of isoforms have sensory defects that prevent them from mating normally [8]. On the other hand, female mice presenting the deletion of exons 2 and 3 of the gene mate normally, but have oocytes with reduced developmental competence, having an increased frequency of spindle defects in ovulated oocytes, reduced follicular pool size and poor oocyte quality [9], being similar to the set of embryonic defects observed in patients undergoing IVF, particularly older patients [10]. The expression of Tp73 in mice oocytes also declines with natural aging [9], suggesting that it is involved in maintaining genomic stability in oocytes. Steuerwald and collaborators [11] have also seen this down regulation of p73 transcripts in oocyte of older women patients. Another study with women submitted to in vitro fertilization (IVF) treatment showed an association of one single nucleotide polymorphism–SNP (rs4648551, A>G) on the TP73 gene with infertility, exhibiting a clear enrichment of the G allele in older infertile women (>35 years) [12]. Others studies have related the use of SNPs as possible markers to infertility problems [13–15].

Given the importance of this gene in fertility and the possible use of the polymorphisms as a marker to help treatment, we examined the association between one polymorphism on the TP73 gene (rs4648551, A>G) and the following parameters related with ovarian reserve: age, antral follicles count (AFC), anti-Müllerian hormone (AMH) levels and the ovarian response prediction index (ORPI = (AMH x AFC)/patient age) [16, 17]. The use of this polymorphism as a marker can be of interest as a potential prognostic factor in cases of human female infertility and could contribute to the improvement in the diagnosis of subfertility and may facilitate personalized strategies in the infertility therapy.

## Materials and Methods

### Patients

Our study included blood samples of 605 Brazilian women attending their first intracytoplasmic sperm injection (ICSI) cycle between February 2012 and June 2014. The criteria for inclusion were the following: age ≤ 37 years, body mass index (BMI) between 20–30 kg/m^2^, regular menstrual cycles, both ovaries present, no history of ovarian surgery, no severe endometriosis and no evidence for endocrine disorders or ovarian cysts.

In addition, for comparison of population genotype distribution, a total of 83 volunteer that had at least one healthy child with any infertility treatment were included. The study was authorized by the local ethical committee (the *Comitê de Ética em Pesquisa do Centro de Referência da Saúde da Mulher;* project reference: 045/11) and was conducted according to the principles expressed in the Declaration of Helsinki. A written informed consent was obtained from all recruited subjects.

### DNA extraction and Genotyping

Genomic DNA for all subjects was extracted from the sample of blood for each patient using the QIAamp DNA blood mini kit (Qiagen) following the manufacturer’s instructions.

Genotyping was performed in duplicate by real time polymerase chain reaction (PCR) amplification using a TaqMan SNP genotyping assay (C_26892242_10) according to Applied Biosystems standard protocols. Each 10μl reaction composed of 1μl of genomic DNA (100ng/μl), 5μl of UMM (TaqMan Universal Master Mix), 0.5μl probe (rs4648551), and 3.5μl of DNase-free water was performed according to the following amplification protocol: denaturation at 95°C for 10 min, followed by 40 cycles of 92°C for 15s and 60°C for 1min in StepOnePlus Real-Time PCR. Products were analyzed by TaqMan Genotyper Software v1.3 (ABI). Some samples were subjected to sequencing to validate the genotyping results.

### AMH measurement

AMH was measured using an enzymatically amplified 2-site immunoassay kit (AMH Gen II ELISA, Beckman Coulter Inc.) according to the manufacturer’s manual. The lowest detection limit of this assay is 0.01 ng/ml. To minimize the chances of bias in the assay, all sera were processed in duplicate during the same day, using the same measurement kits, and by the same operator. Low and high level controls were included in each assay.

### Antral follicles count

All women had a transvaginal ultrasonographic evaluation performed during the early follicular phase of a previous cycle before the scheduled treatment. A single experienced sonographer, who was blinded to the results of any hormonal assays and the patient’s age, performed the evaluation using a conventional 2-dimensional transvaginal ultrasound at 7 MHz (Medison Digital Color MT; Medison Co. Ltd., Seoul, Korea). The total number of 2–9 mm antral follicles in both ovaries was taken in account.

### Statistical analysis

Statistical analysis was performed using StatsDirect version 2.7.9 software, and the Hardy-Weinberg equilibrium was applied using an online calculator, available on http://ihg.gsf.de. The differences in the frequencies of the SNP genotypes and/or alleles in the study and control groups were evaluated using Fisher’s test. The odds ratio (OR) was calculated by logistic regression. All statistical tests were considered significant at a level of P < 0.05.

## Results

A total of 605 patients and 83 control women were genotyped for the p73 rs4648551 A>G SNP. We found that the genotypes of the study group and control group were similar, having no significant difference in genotype and allelic frequency distributions of p73 rs4648551 A>G SNP (Table 1). All allelic and genotypic frequencies observed during this study are consistent with the Hardy-Weinberg equilibrium.

**Table 1:** Genotypes and alleles frequencies distribution in the control and study groups.

P73 (rs4648551)	SNP Genotype	Control n (%)	Study group n (%)	P
	AA	17 (20.5)	118 (19.5)	
Genotypes	GA	32 (38.5)	274 (45.3)	0.48
	GG	34 (41.0)	213 (35.2)	
Alleles	A	66 (39.8)	510 (42.1)	0.56
	G	100 (60.2)	700 (57.9)	

Analysis of the genotype distribution of the P73 (rs4648551, A>G) polymorphism in the study group, associated with the characters related to ovarian reserve investigated here, revealed a significant difference. Patients that presented the AA genotype had the lowest levels of AMH (P = 0.02), the lowest number of antral follicles (P = 0.01) and the lowest ORPI (P = 0.007). The genotype distribution and the means of AMH levels, AFC and ORPI are presented in Table 2. There were no significant difference between the different genotypes and age.

**Table 2:** Mean of age, AMH level, AFC and ORPI of SNP genotypes in the study group.

P73 (rs4648551)	AA	AG	GG	P
% (n/total)	19.5% (118/605)	45.3% (274/605)	35.2% (213/605)	
Age (years)	33.2±3.1^a^ ^,^ ^b^	32.6±3.2^a^ ^,^ ^c^	32.9±3.1^b^ ^,^ ^c^	0.21
				0.07^a^
				0.25^b^
				0.49^c^
AMH level (ng/mL)	1.7±1.4 ^a^ ^,^ ^b^	2.1±2.1 ^a^ ^,^ ^c^	2.4±2.8 ^b^ ^,^ ^c^	0.02
				0.02^a^
				0.008^b^
				0.49^c^
AFC	12.7±7.4 ^a^ ^,^ ^b^	14.9±8.4 ^a^ ^,^ ^c^	16.0±11.1 ^b^ ^,^ ^c^	0.01
				0.004^a^
				0.009^b^
				0.94^c^
ORPI	0.9±1.3 ^a^ ^,^ ^b^	1.4±2.2 ^a^ ^,^ ^c^	1.9±4.3 ^b^ ^,^ ^c^	0.007
				0.005^a^
				0.003^b^
				0.65^c^

The P value represents the comparison between genotypes:

^a^AA versus AG;

^b^AA versus GG;

^c^AG versus GG.

Considering that there is an association of the investigated SNP with some of the characters analyzed, we decided to investigate the influence of the different genotypes with ovarian reserve. When patients with AA genotype were (isolated from patients with AG and GG genotypes, we observed a significant difference of this polymorphism associated with lower AMH levels (P = 0.006), AFC (P = 0.003) and ORPI (P = 0.002). When patients with the GG genotype were isolated, no significant difference was observed. Table 3 summarizes our results.

**Table 3:** Mean of age, AMH level, AFC and ORPI of associated SNP genotypes in the study group.

P73(rs4648551)	AA	AG+GG	P	GG	AG+AA	P
% (n/total)	19.5% (118/605)	80.5% (487/605)		35.2% (213/605)	64.8% (392/605)	
Age (years)	33.2±3.1	32.7±3.2	0.10	32.9±3.1	32.8±3.2	0.96
AMH (ng/mL)	1.7±1.4	2.3±2.4	0.006	2.4±2.8	2.0±1.9	0.11
AFC	12.7±7.4	15.4±9.7	0.003	16.0±11.1	14.3±8.2	0.27
ORPI	0.9±1.3	1.6±3.3	0.002	1.9±4.3	1.2±2.0	0.13

Based on the fact that AA genotype was associated with lower levels of AMH, AFC and ORPI, we divided the patients into two groups: diminished ovarian reserve (AMH <1ng/mL, AFC ≤9) and normal ovarian reserve (AMH ≥1ng/mL, AFC>9). The cut-off for diminished ovarian reserve of AMH <1ng/ml was used taken account Nardo, La Marca and colleagues [18, 19]. For AFC ≤9, we used the cut-off based on previous researches associating AMH and AFC [20]. Odds Ratio analysis was performed to verify an association of the AA genotype with decreased ovarian reserve. Women presenting the AA genotype had a 2.0-fold increased risk for having AMH <1 and AFC ≤9 (OR 2.0, 95% CI 1.23–3.31, P = 0.005). Analyzing the alleles, we detected an enrichment of the A allele in the group of diminished ovarian reserve (OR 1.4, 95%CI 1.02–1.83, P = 0.04). Results are shown in Table 4.

**Table 4:** Genotypes and alleles frequencies distribution in diminished and normal ovarian reserve groups.

	Diminished ovarian reserve	Normal ovarian reserve	P	OR	95%CI
**Genotype**	AMH<1+AFC≤9*	AMH≥1+AFC>9*			
AA	37.5% (33/88)	62.5% (55/88)	0.005	2.0	1.23–3.31
AG+GG	22.9% (88/385)	77.1% (297/385)			
**Alleles**					
A	29.1% (113/388)	70.9% (275/388)	0.04	1.4	1.02–1.83
G	23.1% (129/558)	76.9% (429/558)			

* In these groups, the population must necessarily have the two inclusion criteria (AMH and AFC).

## Discussion

The p53 family members are involved in many important processes, contributing to cell cycle regulation, transactivation and apoptosis in response to DNA damage [21]. Besides, the members of the p53 family have also been described as regulators of crucial process related to human reproduction [22, 23]. Observations have shown that the p73 plays a crucial role in maintaining the size of the follicular pool and ovulation rate, as well as acting at the spindle checkpoint [12], affecting the oocyte quality [9].

Recent studies have revealed that several genetic polymorphisms may play important roles in human reproduction [4, 13–15]. The present study investigated the association between the p73 rs4648551 A>G polymorphism and the ovarian reserve in Brazilian women undergoing IVF/ICSI treatment. Given that the p73 gene is associated with fertility, small variations in its activity can cause individual differences on the ovarian reserve and can present as potential targets of reproductive disorders. However, little is known about how this polymorphism acts in the fertility of women.

Our results showed a significant association of AMH levels, AFC and ORPI with the different genotypes of the p73 rs4648551 A>G polymorphism. Despite the fact that Feng and colleagues [12] have shown a relationship between this polymorphism and infertile patients with age ≥35 years, we did not observe this association. The lack of any relationship between this polymorphism and age could be explained in part because we did not include women older than 37 years in order to minimize the ageing effect on ovarian reserve.

Our findings showed an association of the AA genotype and lower mean levels of AMH, AFC and ORPI. Tomasini and collegues [9] had already shown an association of this gene and follicular numbers in mice.

The division of the study group in two subgroups: diminished ovarian reserve (AMH<1, AFC≤9) and normal ovarian reserve (AMH≥1, AFC>9) showed more clearly the association of the AA genotype with the first subgroup and an enrichment of the A allele in the same subgroup.

Data regarding treatment outcome (rFSH dosage, rLH dosage, number of retrieved oocytes and implantation, pregnancy and abortion rates) and TP73 gene polymorphism are under analysis for further publication. In conclusion, this study shows that the p73 rs4648551 A>G polymorphism can be involved in the ovarian reserve. Women with AA genotype have 2.0-fold increased risk for having a diminished ovarian reserve. To the best of our knowledge, the present study is the first to analyze this polymorphism in humans for assessing the numbers of ovarian follicles and AMH levels and, therefore, the ovarian reserve. Additional validation is needed to provide more information regarding the use of this polymorphism as a potential ovarian reserve marker.
